# 2,2′-[Biphenyl-2,2′-diylbis(­oxy)]diacetic acid monohydrate

**DOI:** 10.1107/S160053680802833X

**Published:** 2008-09-13

**Authors:** Muhammad Rabnawaz, Qamar Ali, Muhammad Raza Shah, Kuldip Singh

**Affiliations:** a HEJ Research Institute of Chemistry, International Center for Chemical and Biological Sciences, University of Karachi, Karachi 75270, Pakistan; b Department of Chemistry, University of Leicester, George Porter Building, University Road, Leicester LE1 7RH, England

## Abstract

In the crystal structure of the title compound, C_16_H_14_O_6_·H_2_O, the dihedral angle between the benzene rings is 60.8 (3)°. Mol­ecules are linked through a bifurcated O—H⋯O hydrogen bond, forming a zigzag chain along the *b* axis. The chains are further linked by O—H⋯O hydrogen bonds mediated by water mol­ecules.

## Related literature

For the crystal structures of related compounds, see: Ali, Hussain *et al.* (2008 ▶); Ali, Ibad *et al.* (2008 ▶); Ali, Shah & VanDerveer (2008 ▶); Ibad *et al.* (2008 ▶). For biological applications, see: Baudry *et al.* (2006 ▶); Kamoda *et al.* (2006 ▶); Litvinchuk *et al.* (2004 ▶); MacNeil & Decken (1999 ▶); Park (2000 ▶); Sisson *et al.* (2006 ▶).
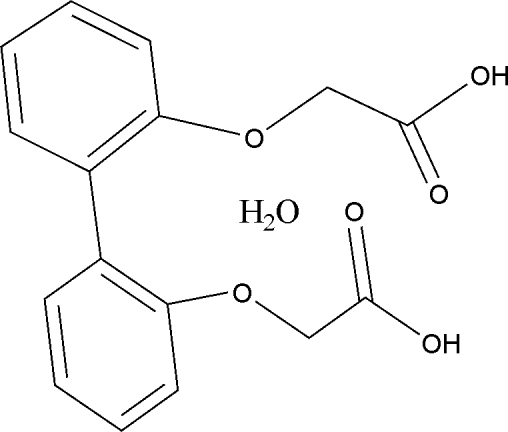


## Experimental

### 

#### Crystal data

C_16_H_14_O_6_·H_2_O
*M* *_r_* = 320.29
Monoclinic, 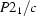

*a* = 13.7590 (17) Å
*b* = 6.7875 (9) Å
*c* = 16.446 (2) Å
β = 104.698 (2)°
*V* = 1485.6 (3) Å^3^
*Z* = 4
Mo *K*α radiation
μ = 0.11 mm^−1^
*T* = 150 (2) K
0.17 × 0.13 × 0.08 mm

#### Data collection

Bruker APEX 2000 CCD area-detector diffractometer
Absorption correction: multi-scan (*SADABS*; Sheldrick, 1996 ▶) *T*_min_ = 0.981, *T*_max_ = 0.991
10352 measured reflections
2612 independent reflections
1555 reflections with *I* > 2σ(*I*)
*R*_int_ = 0.078

#### Refinement

*R*[*F* ^2^ > 2σ(*F* ^2^)] = 0.048
*wR*(*F* ^2^) = 0.088
*S* = 0.85
2612 reflections
216 parameters
3 restraints
H atoms treated by a mixture of independent and constrained refinement
Δρ_max_ = 0.16 e Å^−3^
Δρ_min_ = −0.17 e Å^−3^

### 

Data collection: *SMART* (Bruker, 1997 ▶); cell refinement: *SAINT* (Bruker, 1997 ▶); data reduction: *SAINT*; program(s) used to solve structure: *SHELXS97* (Sheldrick, 2008 ▶); program(s) used to refine structure: *SHELXL97* (Sheldrick, 2008 ▶); molecular graphics: *SHELXTL* (Sheldrick, 2008 ▶); software used to prepare material for publication: *SHELXTL*.

## Supplementary Material

Crystal structure: contains datablocks I, global. DOI: 10.1107/S160053680802833X/is2319sup1.cif

Structure factors: contains datablocks I. DOI: 10.1107/S160053680802833X/is2319Isup2.hkl

Additional supplementary materials:  crystallographic information; 3D view; checkCIF report

## Figures and Tables

**Table 1:** Hydrogen-bond geometry (Å, °)

*D*—H⋯*A*	*D*—H	H⋯*A*	*D*⋯*A*	*D*—H⋯*A*
O3—H3⋯O5^i^	0.84	1.87	2.671 (2)	158
O3—H3⋯O4^i^	0.84	2.66	3.285 (2)	133
O6—H6⋯O7^ii^	0.84	1.76	2.584 (3)	165
O7—H7*A*⋯O2	0.833 (16)	2.30 (2)	2.958 (3)	136 (2)
O7—H7*B*⋯O1	0.820 (16)	2.50 (2)	3.153 (3)	138 (3)

Symmetry codes: (i) 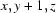; (ii) 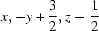.

**Fig. 1.**
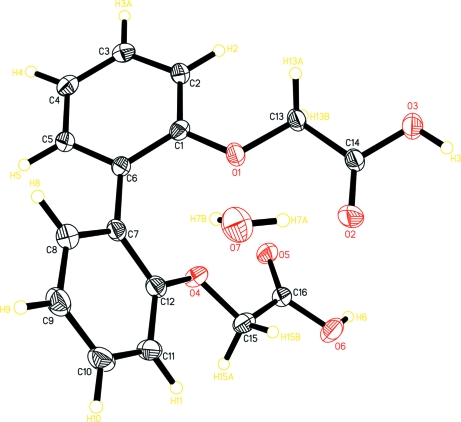
The molecular structure of the title compound. Displacement ellipsoids are drawn at the 30% probability level.

**Fig. 2.**
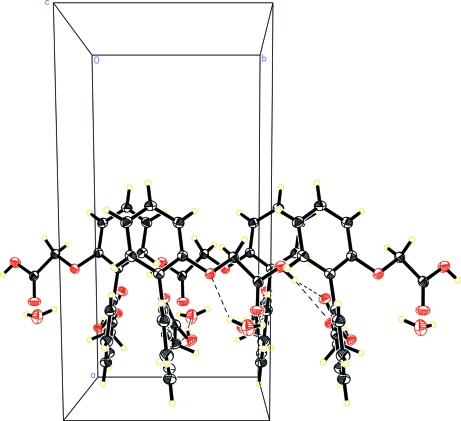
Packing diagram, viewed along the c axis.

## supplementary crystallographic information

### Comment

By virtue of their role as pharmacophore, the biphenyl structural motif is found to be an integral part of several important biologically active compounds (Kamoda *et al.*, 2006). The biphenyl system have been extensively studied by X-ray crystallography (Ali, Hussain *et al.*, 2008; Ali, Ibad *et al.*, 2008; Ali, Shah & VanDerveer, 2008; Ibad *et al.*, 2008) and their role have been elaborated in the domain of photophysics (Park, 2000). Our interest in the chemistry of biphenyl compounds stems from their use as starting material for the synthesis of oligo(*p*-phenylene)s (Sisson *et al.*, 2006; Litvinchuk *et al.*, 2004). The cylindrical self-assembly of suitably substituted oligo(*p*-phenylene)s result in the formation of functionalized pores (Baudry *et al.*, 2006). We report now the synthesis and crystal structure of 2,2'-[biphenyl-2,2'-diylbis(oxy)]diacetic acid.

The dihedral angle between the planes of the benzene rings is 60.8 (3)°. The inter ring C6—C7 distance is 1.488 (3) Å, which compares well with the reported value (MacNeil & Decken 1999). The molecules of the title compound (Fig. 1) are interacting through intermolecular and intramolecular hydrogen bonding (Table 1) involving carbonyl and hydrogen of the type C═O···HO (Fig. 2). The intermolecular hydrogen bonding is mediated by water molecules (Fig. 2). The two carboxylic acid groups are oriented in the same directions (Fig. 1) due to hydrogen bonding contrary to its hydrazide (Ibad *et al.*, 2008) and ester analogue (Ali, Hussain *et al.*, 2008; Ali, Ibad *et al.*, 2008; Ali, Shah & VanDerveer, 2008) where both carboxylic acids are oriented in different directions. The C14—O2 and C16—O5 distances in the title compound are 1.207 (5) and 1.194 (5) Å, respectively, typical of double bonds (Ibad *et al.*, 2008). The OCH_2_COOH substituent are having torsion angle 165.3 (4)° (C13—O1—C1—C6) and 178.6 (4)° (C15—O4—C12—C7) with respect to phenyl rings.

### Experimental

The title compound 1 was synthesized in two steps. In the first step *tert*-butyl 2-({2'-[2-(*tert*-butoxy)-2-oxoethoxy][1,1'-biphenyl]-2- yl}oxy) acetate (compound 2) was prepared and in the second step the *tert*-butyl group was removed to obtain the title compound 1. The experimental procedure of the both steps is presented below. 1. Synthesis of *tert*-butyl 2-({2'-[2-(*tert*-butoxy)-2-oxoethoxy][1,1'-biphenyl] -2- yl}oxy)acetate 2: K_2_CO_3_ (414 mg, 3 mmol) and 2,2'-dihydroxybiphenyl (186 mg, 1 mmol) in 15 ml of acetone were stirred for 10 minutes, followed by addition of tertiary butyl bromoacetate (371 mg, 3 mmol). The reaction mixture was stirred at room temperature for three hours. Solvent was evaporated under reduced pressure and the residue was dissolved in a mixture of water (50 ml) and dichloromethane (50 ml). The aqueous layer was extracted three times with dichloromethane.The combined organic phases were evaporated under reduced pressure and the solid residue was dissolved in hot hexane and slow evaporation of hot hexane give us colorless crystals (736 mg) in 80% yield. 2. Synthesis of 2,2'-[biphenyl-2,2'-diylbis(oxy)]diacetic acid 1: 500 mg (1.2 mmol) of was dissolved in 10 ml of dichloromethane and 5 ml of TFA was added to the stirred solution. The reaction was monitor with thin layer chromatography (hexane:choroform 2:8) after each 10 minutes of interval. After 30 minutes the starting material disappeared. All the TFA and solvent were removed through freeze drying and the solid residue was dissolved in methanol, slow evaporation of methanol at room temperature yielded colorless crystals (310 mg, 85%).

### Refinement

Water H atoms were located in a difference Fourier map and were refined with distance restraints of O—H = 0.83 (2) and H···H = 1.35 (2) Å. Other H atoms were placed at calculated positions (C—H = 0.95 - 0.99 and O—H = 0.84 Å) and were treated as riding, with *U*_iso_(H) = 1.2*U*_eq_(C) or 1.5*U*_eq_(O).

### Crystal data

**Table:**

C_16_H_14_O_6_·H_2_O	*F*(000) = 672
*M**_r_* = 320.29	*D*_x_ = 1.432 Mg m^−^^3^
Monoclinic, *P*2_1_/*c*	Mo *K*α radiation, λ = 0.71073 Å
Hall symbol: -P 2ybc	Cell parameters from 765 reflections
*a* = 13.7590 (17) Å	θ = 2.6–23.3°
*b* = 6.7875 (9) Å	µ = 0.11 mm^−^^1^
*c* = 16.446 (2) Å	*T* = 150 K
β = 104.698 (2)°	Block, colourless
*V* = 1485.6 (3) Å^3^	0.17 × 0.13 × 0.08 mm
*Z* = 4	

### Data collection

**Table:**

Bruker APEX 2000 CCD area-detector diffractometer	2612 independent reflections
Radiation source: fine-focus sealed tube	1555 reflections with *I* > 2σ(*I*)
graphite	*R*_int_ = 0.078
φω scans	θ_max_ = 25.0°, θ_min_ = 1.5°
Absorption correction: multi-scan (SADABS; Sheldrick, 1996)	*h* = −15→16
*T*_min_ = 0.981, *T*_max_ = 0.991	*k* = −8→8
10352 measured reflections	*l* = −19→19

### Refinement

**Table:**

Refinement on *F*^2^	Primary atom site location: structure-invariant direct methods
Least-squares matrix: full	Secondary atom site location: difference Fourier map
*R*[*F*^2^ > 2σ(*F*^2^)] = 0.048	Hydrogen site location: inferred from neighbouring sites
*wR*(*F*^2^) = 0.088	H atoms treated by a mixture of independent and constrained refinement
*S* = 0.85	*w* = 1/[σ^2^(*F*_o_^2^) + (0.0212*P*)^2^] where *P* = (*F*_o_^2^ + 2*F*_c_^2^)/3
2612 reflections	(Δ/σ)_max_ < 0.001
216 parameters	Δρ_max_ = 0.16 e Å^−^^3^
3 restraints	Δρ_min_ = −0.17 e Å^−^^3^

### Special details

**Table:**

Geometry. All e.s.d.'s (except the e.s.d. in the dihedral angle between two l.s. planes) are estimated using the full covariance matrix. The cell e.s.d.'s are taken into account individually in the estimation of e.s.d.'s in distances, angles and torsion angles; correlations between e.s.d.'s in cell parameters are only used when they are defined by crystal symmetry. An approximate (isotropic) treatment of cell e.s.d.'s is used for estimating e.s.d.'s involving l.s. planes.
Refinement. Refinement of *F*^2^ against ALL reflections. The weighted *R*-factor *wR* and goodness of fit *S* are based on *F*^2^, conventional *R*-factors *R* are based on *F*, with *F* set to zero for negative *F*^2^. The threshold expression of *F*^2^ > σ(*F*^2^) is used only for calculating *R*-factors(gt) *etc*. and is not relevant to the choice of reflections for refinement. *R*-factors based on *F*^2^ are statistically about twice as large as those based on *F*, and *R*- factors based on ALL data will be even larger.

### Fractional atomic coordinates and isotropic or equivalent isotropic displacement parameters (Å^2^)

**Table:**

	*x*	*y*	*z*	*U*_iso_*/*U*_eq_	
O1	0.66330 (12)	0.7028 (2)	0.14229 (10)	0.0345 (5)	
O2	0.77830 (14)	0.9862 (3)	0.10441 (13)	0.0575 (6)	
O3	0.64570 (12)	1.1309 (3)	0.01909 (11)	0.0425 (5)	
H3	0.6876	1.2187	0.0158	0.064*	
O4	0.81532 (12)	0.4297 (2)	0.13157 (10)	0.0358 (5)	
O5	0.74938 (13)	0.4148 (3)	−0.03373 (11)	0.0401 (5)	
O6	0.88580 (13)	0.5688 (3)	−0.05041 (11)	0.0537 (6)	
H6	0.8564	0.5573	−0.1016	0.080*	
C1	0.60904 (18)	0.5402 (4)	0.15417 (15)	0.0291 (6)	
C2	0.50827 (18)	0.5152 (4)	0.11531 (15)	0.0324 (7)	
H2	0.4738	0.6084	0.0752	0.039*	
C3	0.45771 (19)	0.3522 (4)	0.13553 (15)	0.0336 (7)	
H3A	0.3883	0.3352	0.1094	0.040*	
C4	0.50694 (18)	0.2161 (4)	0.19259 (15)	0.0354 (7)	
H4	0.4724	0.1038	0.2053	0.043*	
C5	0.60788 (19)	0.2438 (4)	0.23173 (15)	0.0336 (7)	
H5	0.6415	0.1509	0.2725	0.040*	
C6	0.66082 (18)	0.4036 (4)	0.21279 (15)	0.0281 (6)	
C7	0.76791 (18)	0.4314 (3)	0.25873 (15)	0.0294 (6)	
C8	0.7942 (2)	0.4382 (4)	0.34570 (17)	0.0396 (7)	
H8	0.7430	0.4284	0.3749	0.048*	
C9	0.8932 (2)	0.4588 (4)	0.39121 (17)	0.0445 (8)	
H9	0.9096	0.4634	0.4509	0.053*	
C10	0.9674 (2)	0.4726 (4)	0.34935 (18)	0.0428 (7)	
H10	1.0353	0.4878	0.3803	0.051*	
C11	0.94422 (19)	0.4646 (4)	0.26314 (17)	0.0383 (7)	
H11	0.9962	0.4723	0.2347	0.046*	
C12	0.84508 (19)	0.4451 (4)	0.21760 (16)	0.0319 (6)	
C13	0.61476 (18)	0.8429 (3)	0.08196 (15)	0.0314 (6)	
H13A	0.5600	0.9081	0.1009	0.038*	
H13B	0.5852	0.7769	0.0276	0.038*	
C14	0.6906 (2)	0.9920 (4)	0.07165 (16)	0.0326 (6)	
C15	0.88486 (18)	0.4850 (4)	0.08631 (16)	0.0394 (7)	
H15A	0.9416	0.3906	0.0970	0.047*	
H15B	0.9119	0.6178	0.1039	0.047*	
C16	0.8314 (2)	0.4852 (4)	−0.00470 (17)	0.0352 (7)	
O7	0.81871 (18)	0.9230 (3)	0.28791 (13)	0.0573 (6)	
H7A	0.814 (2)	1.003 (3)	0.2488 (15)	0.086*	
H7B	0.795 (2)	0.818 (3)	0.2670 (18)	0.086*	

### Atomic displacement parameters (Å^2^)

**Table:**

	*U*^11^	*U*^22^	*U*^33^	*U*^12^	*U*^13^	*U*^23^
O1	0.0320 (11)	0.0300 (10)	0.0373 (11)	−0.0022 (9)	0.0012 (8)	0.0097 (9)
O2	0.0260 (11)	0.0528 (13)	0.0867 (17)	−0.0005 (10)	0.0012 (11)	0.0256 (12)
O3	0.0379 (12)	0.0388 (12)	0.0470 (12)	−0.0057 (9)	0.0037 (10)	0.0132 (10)
O4	0.0298 (10)	0.0457 (12)	0.0344 (11)	−0.0067 (9)	0.0127 (9)	−0.0042 (9)
O5	0.0323 (11)	0.0433 (12)	0.0435 (12)	−0.0070 (10)	0.0076 (9)	0.0037 (9)
O6	0.0405 (12)	0.0756 (15)	0.0449 (13)	−0.0172 (11)	0.0108 (10)	0.0141 (12)
C1	0.0306 (15)	0.0295 (16)	0.0297 (16)	−0.0015 (13)	0.0124 (13)	−0.0012 (13)
C2	0.0333 (16)	0.0354 (16)	0.0282 (16)	0.0033 (13)	0.0069 (13)	0.0061 (13)
C3	0.0268 (16)	0.0404 (17)	0.0340 (17)	−0.0015 (14)	0.0082 (13)	−0.0039 (14)
C4	0.0329 (17)	0.0366 (17)	0.0399 (18)	−0.0051 (14)	0.0151 (14)	−0.0011 (14)
C5	0.0333 (17)	0.0367 (17)	0.0332 (16)	0.0060 (13)	0.0128 (13)	0.0077 (13)
C6	0.0293 (15)	0.0315 (16)	0.0248 (15)	0.0030 (13)	0.0096 (12)	0.0026 (13)
C7	0.0311 (16)	0.0245 (15)	0.0312 (16)	0.0016 (12)	0.0057 (13)	0.0043 (12)
C8	0.0394 (18)	0.0409 (18)	0.0387 (18)	0.0030 (14)	0.0101 (14)	0.0079 (14)
C9	0.0443 (19)	0.0484 (19)	0.0352 (18)	0.0009 (16)	−0.0004 (15)	0.0086 (15)
C10	0.0321 (16)	0.0431 (19)	0.0453 (19)	0.0002 (15)	−0.0048 (14)	0.0042 (15)
C11	0.0277 (16)	0.0381 (17)	0.0480 (19)	0.0023 (14)	0.0079 (14)	0.0029 (14)
C12	0.0323 (16)	0.0286 (16)	0.0336 (17)	0.0022 (13)	0.0061 (13)	0.0028 (13)
C13	0.0332 (16)	0.0282 (15)	0.0306 (16)	0.0018 (13)	0.0039 (13)	0.0071 (13)
C14	0.0362 (17)	0.0300 (16)	0.0320 (17)	0.0057 (14)	0.0094 (13)	0.0074 (13)
C15	0.0295 (15)	0.0489 (19)	0.0411 (18)	−0.0045 (14)	0.0113 (14)	0.0051 (14)
C16	0.0322 (16)	0.0340 (17)	0.0416 (18)	0.0028 (14)	0.0134 (14)	0.0062 (14)
O7	0.0706 (15)	0.0440 (13)	0.0565 (15)	0.0038 (13)	0.0144 (13)	−0.0040 (11)

### Geometric parameters (Å, °)

**Table:**

O1—C1	1.374 (3)	C6—C7	1.488 (3)
O1—C13	1.414 (3)	C7—C8	1.384 (3)
O2—C14	1.191 (3)	C7—C12	1.399 (3)
O3—C14	1.322 (3)	C8—C9	1.386 (3)
O3—H3	0.8400	C8—H8	0.9500
O4—C12	1.374 (3)	C9—C10	1.371 (3)
O4—C15	1.404 (3)	C9—H9	0.9500
O5—C16	1.207 (3)	C10—C11	1.373 (3)
O6—C16	1.317 (3)	C10—H10	0.9500
O6—H6	0.8400	C11—C12	1.386 (3)
C1—C2	1.382 (3)	C11—H11	0.9500
C1—C6	1.396 (3)	C13—C14	1.494 (3)
C2—C3	1.391 (3)	C13—H13A	0.9900
C2—H2	0.9500	C13—H13B	0.9900
C3—C4	1.367 (3)	C15—C16	1.491 (3)
C3—H3A	0.9500	C15—H15A	0.9900
C4—C5	1.387 (3)	C15—H15B	0.9900
C4—H4	0.9500	O7—H7A	0.833 (16)
C5—C6	1.385 (3)	O7—H7B	0.820 (16)
C5—H5	0.9500		
			
C1—O1—C13	117.58 (19)	C8—C9—H9	120.3
C14—O3—H3	109.5	C9—C10—C11	120.6 (3)
C12—O4—C15	117.41 (19)	C9—C10—H10	119.7
C16—O6—H6	109.5	C11—C10—H10	119.7
O1—C1—C2	123.4 (2)	C10—C11—C12	120.0 (3)
O1—C1—C6	115.6 (2)	C10—C11—H11	120.0
C2—C1—C6	120.9 (2)	C12—C11—H11	120.0
C1—C2—C3	119.4 (2)	O4—C12—C11	124.0 (2)
C1—C2—H2	120.3	O4—C12—C7	115.4 (2)
C3—C2—H2	120.3	C11—C12—C7	120.6 (2)
C4—C3—C2	120.7 (2)	O1—C13—C14	108.4 (2)
C4—C3—H3A	119.7	O1—C13—H13A	110.0
C2—C3—H3A	119.7	C14—C13—H13A	110.0
C3—C4—C5	119.4 (3)	O1—C13—H13B	110.0
C3—C4—H4	120.3	C14—C13—H13B	110.0
C5—C4—H4	120.3	H13A—C13—H13B	108.4
C6—C5—C4	121.5 (2)	O2—C14—O3	124.9 (2)
C6—C5—H5	119.2	O2—C14—C13	125.7 (2)
C4—C5—H5	119.2	O3—C14—C13	109.5 (2)
C5—C6—C1	118.0 (2)	O4—C15—C16	107.7 (2)
C5—C6—C7	119.7 (2)	O4—C15—H15A	110.2
C1—C6—C7	122.2 (2)	C16—C15—H15A	110.2
C8—C7—C12	117.8 (2)	O4—C15—H15B	110.2
C8—C7—C6	119.6 (2)	C16—C15—H15B	110.2
C12—C7—C6	122.6 (2)	H15A—C15—H15B	108.5
C7—C8—C9	121.6 (3)	O5—C16—O6	123.7 (3)
C7—C8—H8	119.2	O5—C16—C15	124.8 (2)
C9—C8—H8	119.2	O6—C16—C15	111.5 (2)
C10—C9—C8	119.4 (3)	H7A—O7—H7B	107 (2)
C10—C9—H9	120.3		
			
C13—O1—C1—C2	−5.2 (3)	C6—C7—C8—C9	178.3 (2)
C13—O1—C1—C6	178.4 (2)	C7—C8—C9—C10	−0.1 (4)
O1—C1—C2—C3	−175.6 (2)	C8—C9—C10—C11	−0.5 (4)
C6—C1—C2—C3	0.5 (4)	C9—C10—C11—C12	0.9 (4)
C1—C2—C3—C4	−0.7 (4)	C15—O4—C12—C11	−16.4 (3)
C2—C3—C4—C5	1.3 (4)	C15—O4—C12—C7	166.3 (2)
C3—C4—C5—C6	−1.7 (4)	C10—C11—C12—O4	−177.9 (2)
C4—C5—C6—C1	1.5 (4)	C10—C11—C12—C7	−0.7 (4)
C4—C5—C6—C7	177.6 (2)	C8—C7—C12—O4	177.5 (2)
O1—C1—C6—C5	175.5 (2)	C6—C7—C12—O4	−0.5 (3)
C2—C1—C6—C5	−0.9 (4)	C8—C7—C12—C11	0.1 (4)
O1—C1—C6—C7	−0.5 (3)	C6—C7—C12—C11	−177.9 (2)
C2—C1—C6—C7	−176.9 (2)	C1—O1—C13—C14	−172.89 (19)
C5—C6—C7—C8	−54.7 (3)	O1—C13—C14—O2	4.7 (4)
C1—C6—C7—C8	121.3 (3)	O1—C13—C14—O3	−176.4 (2)
C5—C6—C7—C12	123.2 (3)	C12—O4—C15—C16	−171.5 (2)
C1—C6—C7—C12	−60.8 (3)	O4—C15—C16—O5	−12.6 (4)
C12—C7—C8—C9	0.3 (4)	O4—C15—C16—O6	168.7 (2)

### Hydrogen-bond geometry (Å, °)

**Table:**

*D*—H···*A*	*D*—H	H···*A*	*D*···*A*	*D*—H···*A*
O3—H3···O5^i^	0.84	1.87	2.671 (2)	158
O3—H3···O4^i^	0.84	2.66	3.285 (2)	133
O6—H6···O7^ii^	0.84	1.76	2.584 (3)	165
O7—H7A···O2	0.83 (2)	2.30 (2)	2.958 (3)	136 (2)
O7—H7B···O1	0.82 (2)	2.50 (2)	3.153 (3)	138 (3)

Symmetry codes: (i) *x*, *y*+1, *z*; (ii) *x*, −*y*+3/2, *z*−1/2.
